# Three-dimensional echocardiography for the evaluation of hypertrophic cardiomyopathy patients: relation to symptoms and exercise capacity

**DOI:** 10.1007/s10554-023-02952-5

**Published:** 2023-10-11

**Authors:** Isabel Cardoso, José Miguel Viegas, Sílvia Aguiar Rosa, Pedro Garcia Brás, André Grazina, Inês Cruz, Luísa Moura Branco, Ana Galrinho, António Fiarresga, Luís R Lopes, Rui Cruz Ferreira

**Affiliations:** 1 Department of Cardiology, Santa Marta Hospital, Rua de Santa Marta, n.50, Lisbon, 1169-024 Portugal; 2 Hospital Garcia de Orta, Almada, Portugal; 3 Inherited Cardiac Disease Unit, Bart’s Heart Centre, St Bartholomew´s Hospital, London, UK; 4 Centre for Heart Muscle Disease, Institute of Cardiovascular Science, University College London, London, UK

**Keywords:** *Hypertrophic cardiomyopathy*, *Left ventricular deformation parameters*, *Three-dimensional speckle-tracking echocardiography*, *Cardiopulmonary exercise testing*, Functional capacity

## Abstract

Patients with hypertrophic cardiomyopathy may exhibit impaired functional capacity, associated with increased morbidity and mortality. Systolic function is one of the determinants of functional capacity. Early identification of systolic disfunction may identify patients at risk for adverse outcomes. Myocardial deformation parameters, derived from three-dimensional (3D) speckle-tracking echocardiography (3DSTE) are useful tools to assess left ventricular systolic function, and are often abnormal before a decline in ejection fraction is seen. The aim of this study was to evaluate the correlation between myocardial deformation parameters obtained by 3DSTE and functional capacity in patients with hypertrophic cardiomyopathy. Seventy-four hypertrophic cardiomyopathy adult patients were prospectively evaluated. All patients underwent a dedicated 2D and 3D echocardiographic examination and cardiopulmonary exercise testing (CPET). Values of 3D global radial (GRS), longitudinal (3DGLS) and circumferential strain (GCS) were overall reduced in our population: 99% (n = 73) of the patients had reduced GLS, 82% (n = 61) had reduced GRS and all patients had reduced GCS obtain by 3DSTE. Average peak VO_2_ was 21.01 (6.08) ml/Kg/min; 58% (n = 39) of the patients showed reduced exercise tolerance (predicted peak VO_2_ < 80%). The average VE/VCO_2_ slope was 29.0 (5.3) and 16% (n = 11) of the patients had impaired ventilatory efficiency (VE/VCO_2_ > 34). In multivariable analysis, 3D GLS (β_1_ = 0.10, 95%CI: 0.03;0.23, p = 0.014), age (β_1_ = -0.15, 95%CI: -0.23; -0.05, p = 0.002) and female gender (β_1_ = -5.10, 95%CI: -7.7; -2.6, p < 0.01) were independently associated with peak VO_2_. No association was found between left ventricle ejection fraction obtain and peak VO_2_ (r = 0.161, p = 0.5). Impaired myocardial deformation parameters evaluated by 3DSTE were associated with worse functional capacity assessed by peak VO_2_.

## Introduction

Hypertrophic cardiomyopathy (HCM) is the most frequent genetic heart disease, with an approximate prevalence of 0.2% [1]. It is characterized by heterogenous left ventricular (LV) hypertrophy and histologically by cardiomyocyte hypertrophy, myocardial fibre disarray and interstitial fibrosis [2].

Patients with hypertrophic cardiomyopathy may exhibit reduced functional capacity, which is associated with increased morbidity and mortality. Progression to heart failure (HF) is a major concern in these patients and can be in part predicted by impaired functional capacity [3].

The pathophysiology of reduced functional capacity in patients with HCM has been mainly linked to left ventricular diastolic and systolic disfunction, chronotropic incompetence and impaired stroke volume response [4].

Cardiopulmonary exercise testing (CPET) is indicated by international guidelines as a safe and reliable test to characterize severity and mechanisms of functional limitation in patients with HCM. Reduced values of peak oxygen, ventilatory efficiency and anaerobic threshold are associated with progression to HF [5].

Systolic function evaluation based on ejection fraction (EF) is not a sensitive method, since the former is typically normal or even increased in this group of patients. Myocardial deformation parameters derived from two-dimension (2D) and three-dimension (3D) speckle tracking echocardiography, on the other hand, are often abnormal before a decline in EF is seen [6].

The heterogeneous LV hypertrophy and interstitial fibrosis that characterize HCM leads to variability in regional and global systolic and diastolic deformation parameters. Characterization and quantification of myocardial segmental and rotational mechanics by 2D and 3D speckle tracking echocardiography (3DSTE) is an evolving and promising technique [2].

3DSTE provides a better quantification of cardiac chambers size and function, minimizing errors provoked by foreshortened views that can occur in standard 2D echocardiography [7].

The aim of this study was to evaluate the association between myocardial deformation parameters obtained by 3DSTE and functional capacity in patients with hypertrophic cardiomyopathy.

## Materials and methods

### Study population

This prospective study included 74 HCM adult patients evaluated at the outpatient cardiomyopathy clinic at Hospital de Santa Marta, Centro Hospitalar Universitário de Lisboa and Hospital Garcia de Orta, Almada between December 2017 and August 2020.

Diagnosis of HCM was made based on the European Society of Cardiology guidelines on Hypertrophic Cardiomyopathy [6]. Briefly a maximal wall thickness (MWT) ≥ 15 mm in probands or ≥ 13 mm in relatives or likely pathogenic variant carriers was considered diagnostic. HCM was considered obstructive if a systolic gradient ≥ 30 mmHg in the left ventricle outflow tract obstruction (LVOT) at rest or after provocative manoeuvre was obtained.

Patients with LV ejection fraction < 50% and LV wall thinning were excluded. Epicardial coronary artery disease and previous septal reduction therapy were also exclusion criteria.

Epicardial coronary artery disease was excluded by invasive coronary angiography or cardiac computerized tomography in symptomatic patients or asymptomatic patients older than 40 years.

The investigation followed the principles outlined in the Declaration of Helsinki. All patients gave written consent.

### Echocardiography

All patients underwent a dedicated 2D and 3D echocardiographic examination (Vivid E95; General Electric). LV morphology and function was assessed according to the recommendations of the American Society of Echocardiography [7]. Evaluation of diastolic function included assessment of transmitral flow pattern with pulse Doppler and tissue Doppler at the mitral annulus, left atrial volume index and maximal velocity of tricuspid regurgitation.

2D global longitudinal strain (GLS) was used to evaluate LV myocardial deformation. Regarding 3D speckle-tracking echocardiography GLS, global circumferential strain (GCS) and global radial strain (GRS) were obtained.

To perform 3D speckle tracking echocardiography (3DSTE) using the acquired full-volume 3D dataset of the left ventricle (LV), the 3D strain analysis software automatically detected the LV’s endocardial and epicardial borders, generating a precise 3D mesh model of the myocardium. Manual adjustments were made by experienced cardiologists. The software utilized “block matching” to compare 3D patterns of acoustic markers within regions of interest (ROIs), identifying and removing outliers. Spatial averaging was then performed for accurate strain analysis, calculating longitudinal, circumferential, and radial strain values for each LV segment throughout the cardiac cycle [8].

2D GLS of -22.5 ± 2.7% was considered normal [9]. Normal 3D values considered were: GLS − 21.0 ± 2.6%, GCS − 30.3 ± 4.0%, GRS 43.2 ± 4.5 [10] [11].

### Cardiopulmonary exercise testing

CPET was performed using a treadmill with the application of the modified Bruce protocol. The duration of the exercise was limited only by patients’ symptoms when reaching maximal effort.

Ventilatory expired gas analysis was performed using Ergostik, Geratherm®, Cardio Solutions systems. The equipment was calibrated in a standard fashion before each test.

A standard 12-lead electrocardiogram (ECG) monitoring, diastolic blood pressure (DBP) and systolic blood pressure (SBP), saturation probe and measure of expiratory gases were recorded.

Oxygen consumption (VO_2_), CO_2_ production (VCO_2_), minute ventilation (VE) and other CPET variables were acquired breath by breath.

Peak VO_2_ (pVO_2_) was measured in each patient and reported as mL kg − 1 min − 1. The expected peak VO_2_ was calculated based on age, gender, and body index (adjusted for body mass/fat-free mass in obese patients) [12].

VE/VCO_2_ at peak exercise was used to obtain the ventilatory class. Anaerobic threshold was measured by V-slope method and by the analysis of ventilatory equivalents.

### Statistical analysis

Data are expressed as mean and standard deviation (SD). The Spearman rank-order correlation coefficient was used to assessed univariable correlation between variables. Variables with significant correlations (p < 0.05) where analyzed using multivariable linear regression. Comparisons of groups were analyzed using 1-way analysis of variance (ANOVA) for parametric data or Kruskal-Wallis for non-parametric data. Analysis within groups was assessed by post-hoc Bonferroni test. A probability value of p < 0.05 was considered significant.

## Results

### Clinical and echocardiographic findings

Of 74 patients with HCM, mean age 56 (15) years, 43 (58%) males, 40 patients (54%) were in New York Heart Association (NYHA) functional class I, 29 (39%) in class II and 5 (7%) in class III. Fifty-five patients (74%) had non obstructive HCM. Maximum LV wall thickness (MWT) was 20 (7) mm. Study population characteristics are shown in Table 1.

**Table 1:** Baseline characteristics of patients with hypertrophic cardiomyopathy

	n = 74
Male gender, n (%)	43 (58)
Age (years), mean (SD)	56 (15)
BSA (m^2^), mean (SD)	1.91 (0.2)
Hypertension, n (%)	38 (51)
Diabetes, n (%)	12 (16)
Dyslipidaemia, n (%)	31 (42)
Current smoker, n (%)	11 (15)
Family history of HCM, n (%)	24 (32)
Beta blocker, n (%)	55 (74)
Calcium channel blocker, n (%)	19 (26)
Angiotensin converting enzyme inhibitors, n (%)	14 (19)
Angiotensin receptor blockers, n (%)	17 (23)
Spironolactone, n (%)	4 (5.4)
Nonobstructive HCM, n (%)	55 (74)
NYHA I, n (%)	40 (54)
NYHA II-III, n (%)	31 (42)
Angina, n (%)	24 (32)
Syncope, n (%)	1 (1.4)
Palpitations, n (%)	24 (32)

Body surface area (BSA), hypertrophic cardiomyopathy (HCM), New York Heart Association (NYHA)

**Fig. 1:**
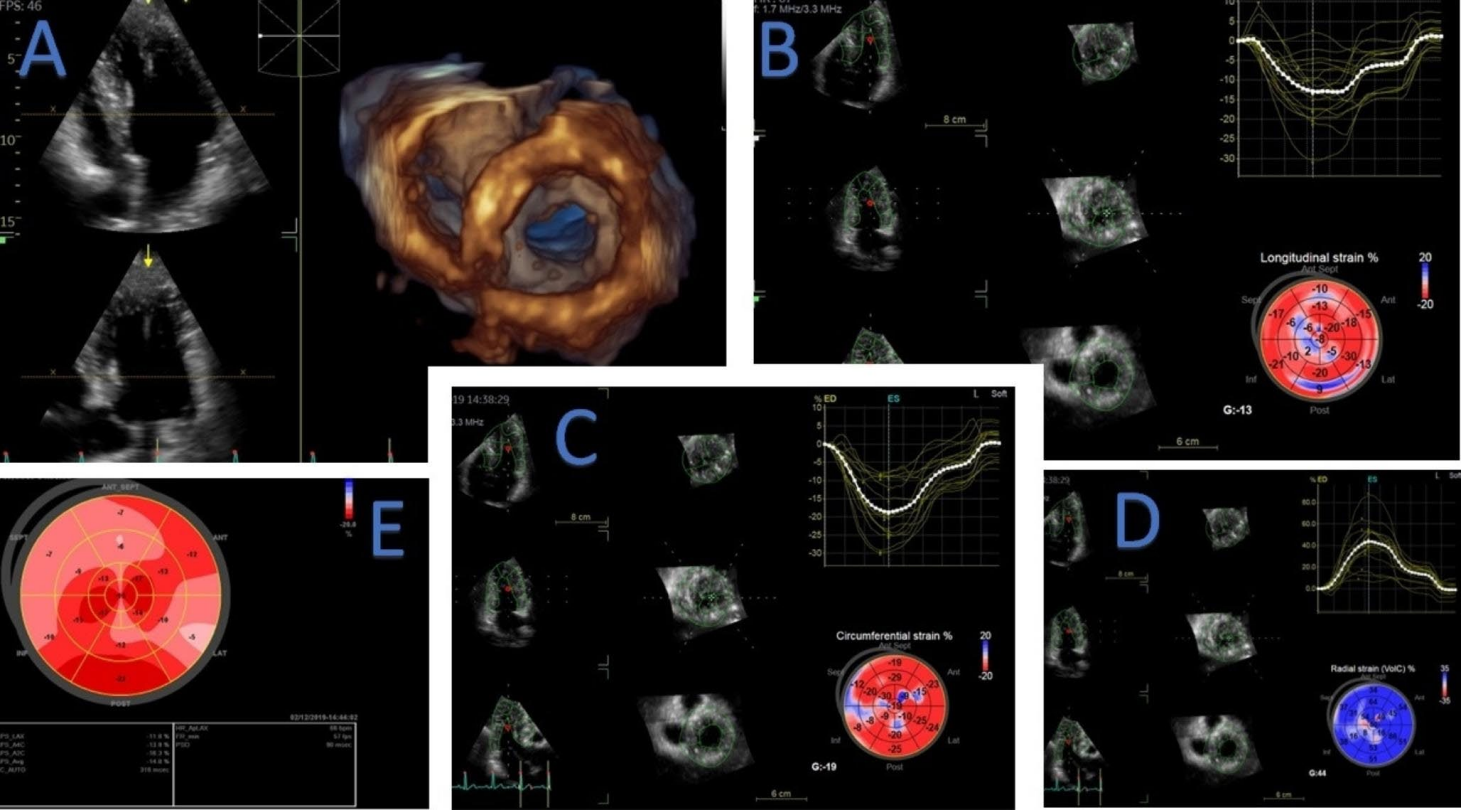
Echocardiographic evaluation of the same patient: 3D echocardiography representation **(A)**, 3D GLS **(B)**, 3D GCS **(C)**, 2D GRS **(D)** and 2D GLS **(E)**

Mean values of global longitudinal, radial, and circumferential strain are reported in Table 2. All patients had reduced 2D GLS. Values of 3D GRS, GLS and GCS were overall reduced in our population – 73 (99%) of the patients had reduced GLS, 61 (82%) had reduced GRS and all patients had reduced GCS obtained by 3DSTE. An example of a echocardiographic study is represented in Fig. 1.

**Table 2:** Echocardiographic findings and cardiopulmonary exercise testing parameters of patients with hypertrophic cardiomyopathy

Echocardiographic parameters		Values, mean (SD)
2D LVEF, mean (SD)		67.5 (7.2)
2D LEVDV, mean (SD)		83.2 (24.6)
2D LESV, mean (SD)		29.5 (12.1)
Global longitudinal strain 2D (%), mean (SD)		-13 (7)
3DLVEF (%), mean (SD)		61.8 (5.9)
Indexed LV mass (g/m2), mean (SD)		97.4 (23.8)
Global longitudinal strain 3D (%), mean (SD)		-10 (4)
Global circumferential strain 3D (%), mean (SD)		-13 (6)
Global radial strain 3D (%), mean (SD)		29 (12)
E- wave (cm/s), mean (SD)		80 (22)
A – wave (cm/s), mean (SD)		69 (25)
E/A ratio, mean (SD)		1.37 (0.89)
Deceleration time (ms), mean (SD)		187 (75)
e’ septal (cm/s), mean (SD)		6 (2)
e’ lateral (cm/s), mean (SD)		8 (3)
a’ septal (cm/s), mean (SD)		8 (3)
a’ lateral (cm/s), mean (SD)		8 (4)
Average E/e’ ratio, mean (SD)		12.8 (4.8)
peak velocity of TR jet (cm/s), mean (SD)		24 (38.6)
LAiVol (ml/m^2)^, mean (SD)		47.27 (16.2)
RV-RA gradient (mmHg), mean (SD)		24 (10)
CPET data		
pVO_2_ (ml/kg/min), mean (SD)		21 (6.67)
% of max predicted VO_2_ (%), mean (SD)		86 (23)
VE/VCO_2_ slope, mean (SD)		29 (5.3)
Time to AT (min), mean (SD)		6 (6.0)
VO_2_ in AT (ml/Kg/min), mean (SD)		14 (4)
Optimal point of ventilation, mean (SD)		25 (5)
RER, mean (SD)		1.05 (0.1)
Time of exercise (min), mean (SD)		12.4 (4.3)

Anaerobic threshold (AT), cardiopulmonary exercise testing (CPET), deceleration time (DT), left atrial index volume (LAiVol), 2D Left ventricle end diastolic volume (2D LVEDV), 2D left ventricle end systolic volume (2DLVESV), Left ventricular ejection fraction (LVEF), peak oxygen consumption (pVO2), minute ventilation/carbon dioxide production (VE/VCO2), right ventricle-right atrium (RV-RA) gradient, respiratory exchange ratio (RER), tricuspid regurgitation (TR).

**Fig. 2:**
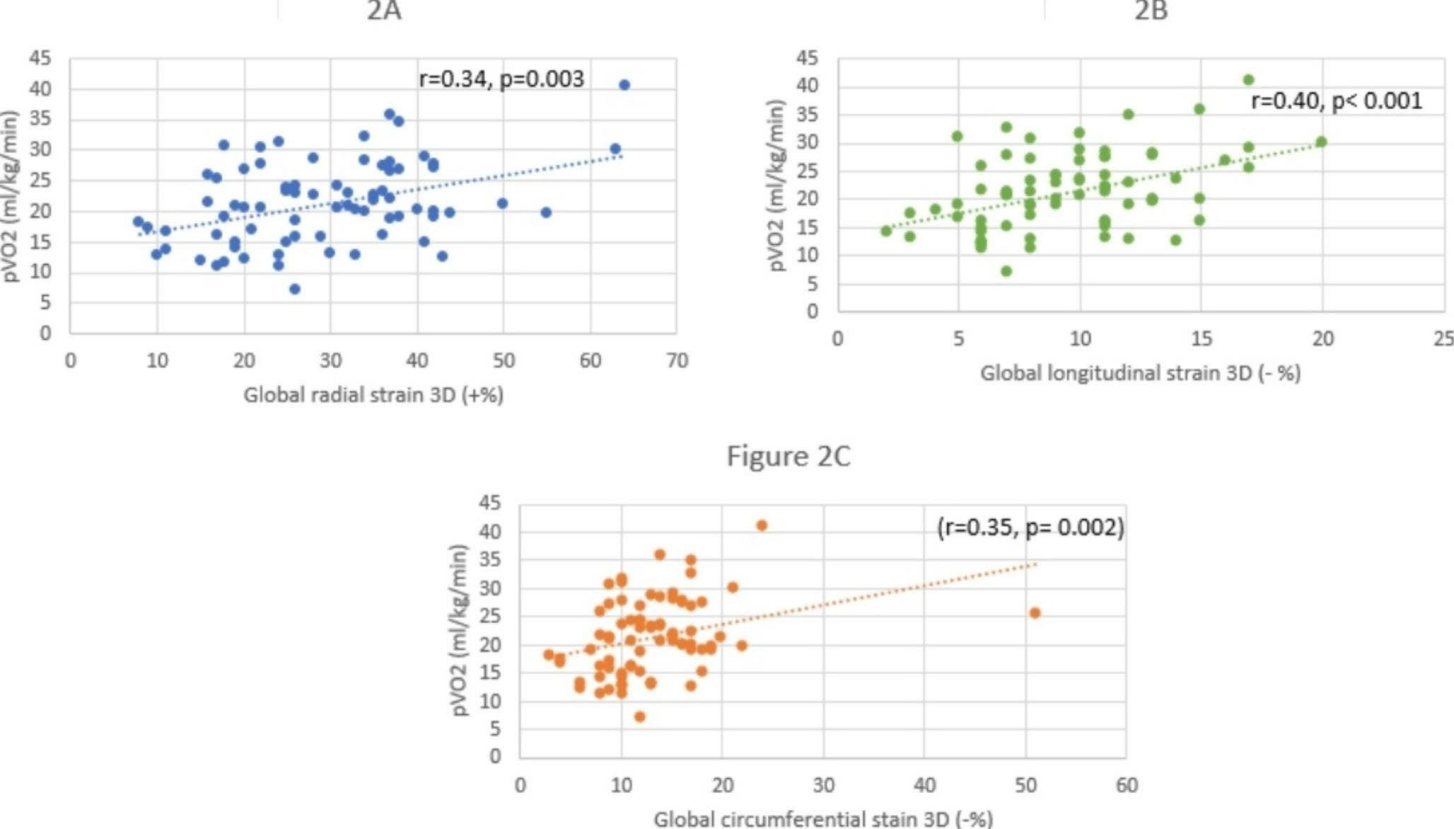
Scatter plot showing the correlation between 3D global radial (**1A**), longitudinal (**1B**) and circumferential strain (**1C**) with peak VO_2_.

Regarding the basal characteristics of our population, an association between NYHA class and 3D GLS was found (F (2.71) = 4.07; p = 0.02). Post hoc analysis with the Bonferroni test revealed that 3D GLS values were significantly lower in patients in NYHA III compared with patients in NYHA I (-4.75 (95% CI, 0.66–8.84%, p = 0.01), but not between patients in NYHA III compared to patients NYHA II (-4.08, CI, -8.26-0.09, p = 0.057) (Fig. 2). The remaining 3D strain measures had no correlation with NYHA class: 3D GRS (F (2,71) = 1.22; p = 0.30) and 3D GCS (F (2,71) = 1.38, p = 0.26).

A relationship between 2D GLS and NYHA class was also found (F (2) = 7.7; p = 0.001).

MWT was not related with 3DSTE strain measures (3D GLS: β_1_ = -0.14, 95%IC -0.35;0.06, p = 0.12; 3D GRS: β_1_ = -0.28, 95%IC -0.94;0.37, p = 0.39; 3D GCS: β_1_ = -0.10, 95%IC -0.45;0.25, p = 0.57), neither was left ventricular outflow tract obstruction (β_1_ = -0.002, 95%IC -0.02;0.02, p = 0.80).

### Cardiopulmonary exercise test findings

Regarding CPET findings respiratory exchange ratio was 1.03 (0.09) revealing adequate exercise effort. Mean time of exercise was 12.4 (4.3) minutes. Average pVO_2_ was 21.01 (6.08) ml/Kg/min; 39 (58%) showed reduced exercise tolerance (predicted pVO_2_ < 80%) and 31 (42%) had a pVO_2_ < 20 ml/Kg/min. The average VE/VCO_2_ slope was 29 (5.3) and 11 (16%) of the patients had impaired ventilatory efficiency (VE/VCO_2_ > 34). CPET findings are shown in Table 2.

There was an association between pVO_2_ and gender (t (72) = 5.52, p < 0.01), pVO_2_ was higher in males – mean 24 (5.8) vs. 17 (5.3) ml/Kg/min than in females.

Regarding the echocardiographic findings, E/e’ showed a inverse relation with pVO_2_ (β_1_ = -0.05, 95%IC -0.9;0.3, p < 0.01) and so did RV-RA peak velocity of tricuspid regurgitant jet (β_1_ = -0.06, 95%IC -0.09;0.01, p = 0.01), however no association was found after adjusting for baseline characteristics in multivariable analysis (p = 0.34; p = 0.25) (Table 3). No association was found between left atrium index volume and pVO_2_ (β_1_ = -0.09, 95%IC -0.2;0.004, p = 0.06).

**Table 3:** Multivariable linear regression for predicting peak VO_2_. Blue shaded cells represent significant p-values

	Multivariable analysis	
	Peak VO_2_	
	Β-estimate	p-value
3D GLS	0.10	0.014
3D GCS	0.17	0.10
3D GRS	0.21	0.05
Age	-0.15	0.002
Female gender	-5.1	< 0.001
E/e’ average	-0.08	0.34
Peak RV-RA gradient	-0.42	0.25

Three-dimension (3D), Global longitudinal strain (GLS), global radial strain (GRS), global circumferential strain (GCS), peak oxygen consumption (pVO_2_), RV-RA (right ventricular – right atrium)

No association was found between pVO_2_ and MWT (β_1_ = 0.024, 95%IC -0.35;0.39, p = 0.10) or between the presence of obstruction and pVO_2_ (t (-65) = − 1.11, p = 0.83).

**Fig. 3:**
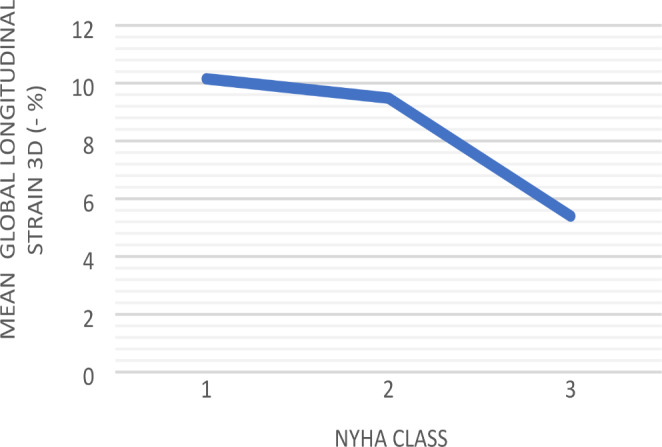
Association between GLS 3D and NYHA class assessed by 1-way ANOVA analysis

Univariable analysis revealed a correlation between 3D GRS (r = 0.34, p = 0.003), 3D GLS (r = 0.40, p < 0.001) and 3D GCS (r = 0.35, p = 0.002) with pVO_2_. On the other hand, no correlation was found between LVEF (r = 0.161, p = 0.5) or GLS (r = 0.169, p = 0.5) obtained by 2D method and pVO_2_ (Fig. 3). No correlation was found between 3D LVEF and pVO_2_ (r=-0.1, p = 0.40).

In multivariable analysis, 3D GLS (β_1_ = 0.1, 95%CI: 0.03;0.23, p = 0.014), age (β_1_ = -0.15, 95%CI: -0.23; -0.05, p = 0.002) and female gender (β_1_=-5.1, 95%CI: -7.7; -2.6, p < 0.01) were independently associated with pVO2. The remaining 3D strain parameters (GRS and GCS) were not considered significantly related to pVO2 in multivariable analysis.

## Discussion

In this study we found that that 3DGLS was independently associated with worse functional capacity determined by peak VO_2,_ 3DGLS was also significantly decreased in patients with higher NYHA classes.

The high variability of left ventricular hypertrophy and contractile capacity between different segments of the myocardium of a single HCM patient, makes 3DSTE a more sensitive method than 2D LVEF to characterize systolic function, considering its higher accuracy at assessing regional cardiac mechanisms. In our study we found a correlation between 3D evaluation of myocardial deformation and functional capacity, which could not be achieved with 2D LVEF.

Overall strain parameters were reduced in our population, as expected for a cohort of HCM patients [13]. Regarding GCS, it was diminished in all the patients of our cohort. Previous studies described an initial compensatory increase in GCS compared do GLS, which is later lost due to disease progression, culminating in an overall fall of strain parameters, which we verified in our cohort [14]. Furthermore, GCS reflects changes in the middle layer of the myocardium where fibres are oriented in a circumferential pattern, as opposed to the inner longitudinal and outer oblique myocardium. Hypertrophy and fibrosis mainly take place in this middle layer, which might explain the overall reduction of this measure verified in our population [15].

As hypothesized, impaired myocardial deformation parameters evaluated by 3DSTE, namely 3D GLS, were associated with worse functional capacity objectively assessed by CPET parameters. On the other hand, there was not a significant correlation between LVEF or GLS obtain by 2D method and pVO_2_.

Absolute values of 3D GLS showed the strongest correlation with pVO_2_ in univariate analysis and were independently associated with pVO2 in multivariable analysis.

3D GLS was also significantly decreased from patients in NYHA class I to class III, which further confirms the association of this parameter with functional capacity. On the other hand, significant GLS variations between patients in NYHA class II and III could not be found. However, 2DGLS was also associated with NYHA class changes.

3D speckle tracking echocardiography has been recognized has a useful tool in the global evaluation of HCM: some studies have reported its utility in predicting atrial fibrillation [13], family screening for HCM [14] and arrhythmic risk stratification [15]. Our findings suggest that 3DSTE might have additional applications in predicting functional capacity.

Incorporating 3DSTE in daily clinical practice can have positive prognostic implications in these patients, allowing for an early identification of patients with worse outcomes, facilitating more effective management strategies and timely interventions. Furthermore, prediction of functional capacity through 3DSTE can represent an alternative for patients who cannot undergo CPET.

### Diastolic disfunction and functional capacity

Our population showed reduced early diastolic mitral annular velocities measured using Doppler tissue imaging. Myocardial hypertrophy, ischemia secondary to coronary microvascular dysfunction and interstitial fibrosis causing chamber stiffness are responsible for impaired ventricular myocardial relaxation, largely described in patients with HCM [6], with consequent increase in intracavitary pressure.

In our cohort average E/e’ ratio correlated with functional capacity in univariate analysis. Unlike conventional Doppler indices alone, the transmitral E to e’ ratio has been particularly correlated with NYHA functional class in patients with HCM [16].

Exercise intolerance in patients with HCM can be, at least in part, attributed to raised left atrial pressures. However, the relation between average E/e’ and left atrium index volume and pVO_2_ were not significant when applied a multivariate analysis, which may indicate that other mechanisms such as reduced stroke volume response, ventilation/perfusion mismatch and abnormal peripheral oxygen utilisation may also influence exercise capacity [17].

Controversy remains regarding left ventricular diastolic pressure and functional capacity at rest as major determinants of exercise capacity in patients with HCM, and more investigation is needed to clear the true mechanisms.

### Study limitations

The present work has some limitations. The sample size is relatively small, which may limit the generalisation of these findings and contributed for the absence of strong correlations in our analysis. Echocardiographic parameters were obtained at rest and not simultaneously with the physical effort.

## Conclusions

Impaired myocardial deformation parameter 3DGLS was independently associated with worse functional capacity assessed by CPET.

Our study supports the emerging data about 3DSTE applications in HCM and its advantages over standard echocardiographic methods, namely in the evaluation of systolic function.

## Abbreviations

CMR: Cardiovascular magnetic resonance
CPET: Cardiopulmonary exercise testing
GCS: Global circumferential strain
GLS: Global longitudinal strain
GRS: Global radial strain
HCM: Hypertrophic cardiomyopathy
LV: Left ventricular
LVOT: Left ventricular outflow tract
MWT: Maximum wall thickness
NYHA: New York Heart Association
NSVT: Non-sustained ventricular tachycardia
TAPSE: Tricuspid annular plane systolic excursion
VCO2: CO2 production
VE: Ventilation
VO_2_: Oxygen uptake
2D: Two-dimensional
3D: Three-dimensional
